# Emerging Insights into Hereditary Alpha-Tryptasemia in the Context of Mast Cell Disorders: A Greek Case Series

**DOI:** 10.3390/jpm16040196

**Published:** 2026-04-01

**Authors:** Fotios Koliofotis, Natalia Katrachoura, Niki Papapostolou, Styliani Taka, Maria Martinou, Anthi Bouchla, Sotirios G. Papageorgiou, Michael Makris

**Affiliations:** 1 National Center of Expertise for Mastocytosis and Mast Cell Disorders, 2nd Department of Dermatology and Venereology, University General Hospital “Attikon”, National and Kapodistrian University of Athens, 12462 Athens, Greece; fkoliofotis.allergy@gmail.com (F.K.); natalia.katrachoura@gmail.com (N.K.); sotpapage@med.uoa.gr (S.G.P.); 2 Allergy Unit, 2nd Department of Dermatology and Venereology, University General Hospital “Attikon”, National and Kapodistrian University of Athens, 12462 Athens, Greece; nikipapap@gmail.com; 3 Allergy Department, 2nd Pediatric Clinic, National and Kapodistrian University of Athens, 11527 Athens, Greece; staka@uoa.gr (S.T.); marymartinou@yahoo.gr (M.M.); 4 Hematology Unit, 2nd Propaedeutic Department of Internal Medicine, University General Hospital “Attikon”, National and Kapodistrian University of Athens, 12462 Athens, Greece; ampouchla@med.uoa.gr

**Keywords:** hereditary alpha tryptasemia, *TPSAB1*, mast cell activation syndrome, systemic mastocytosis, basal serum tryptase, anaphylaxis, mast cell disorders

## Abstract

**Background/Objectives**: Hereditary alpha-tryptasemia (HαT) is increasingly recognized as a genetic modifier of mast cell-mediated disease severity and has been associated with heightened mediator-related symptoms and an elevated risk of anaphylaxis. This study aimed to describe the clinical characteristics, multisystem manifestations, and treatment responses of eight patients with HαT and concomitant mast cell disorders. **Methods**: In this single-center retrospective study, eight adults with confirmed *TPSAB1* copy number gain and a diagnosis of systemic mastocytosis (SM), cutaneous mastocytosis (CM), or mast cell activation syndrome (MCAS) were evaluated. Baseline assessments included demographics, clinical history, basal serum tryptase (BST), *TPSAB1* genotyping, *KIT* D816V testing, and bone marrow examination when indicated. Symptom burden was quantified at baseline and week 8 using the Mastocytosis Activity Score (MAS). All patients received mediator-targeted therapy; omalizumab was administered in selected high-risk cases. **Results**: Eight patients (62.5% male, mean age 53.9 ± 12.0 years) carried *TPSAB1* duplication. The median BST was 16.2 ng/mL (range, 14.3–51.2). Severe anaphylaxis occurred in 75% of patients, predominantly drug-induced, while Hymenoptera venom triggered the remaining cases. Gastroesophageal reflux (87.5%), cutaneous symptoms (62.5%), neuropsychiatric features (62.5%), and autonomic dysfunction (37.5%) were common. The mean MAS decreased significantly from 27.25 ± 7.40 to 18.25 ± 6.48 after 8 weeks of high-dose antihistamines, with omalizumab providing marked additional benefit in selected patients. **Conclusions**: In this cohort, patients with HαT and coexisting mast cell disorders exhibited a high burden of mediator-related symptoms and a notable frequency of anaphylaxis. *TPSAB1* genotyping may provide additional genetic information that aids in contextualizing clinical heterogeneity and mediator-related symptom burden in patients with mast cell disorders. Incorporation of HαT testing into routine evaluation may optimize individualized management.

## 1. Introduction

Hereditary alpha-tryptasemia (HαT) is an autosomal dominant genetic trait resulting from increased germline copy number variations in the *TPSAB1* gene, which encodes for alpha-tryptase, a serine protease predominantly produced and secreted by mast cells [1]. With an estimated prevalence of 5–7% in Western populations, HαT is recognized as the leading cause of persistently increased basal serum tryptase (BST) [2].

Recent studies have demonstrated that HαT is more prevalent among patients with mastocytosis (12–17%) and idiopathic anaphylaxis (IA) (17%) [3]. The clinical spectrum of HαT is highly heterogeneous, ranging from asymptomatic carriers to patients manifesting pronounced clinical symptomatology with extensive multisystemic involvement and functional impairment. HαT has been implicated in the pathophysiology of anaphylactic reactions, cutaneous symptoms, gastrointestinal disorders, neuropsychiatric manifestations, joint hypermobility and connective tissue abnormalities, as well as autonomic nervous system dysfunction [1,4]. The severity of symptoms in patients with HαT correlates positively with the number of *TPSAB1* gene copies, suggesting a gene-dose effect [5,6].

Mastocytosis represents a hematologic neoplasm characterized by excessive accumulation and activation of neoplastic mast cells in various tissues [7]. Mastocytosis is divided into cutaneous mastocytosis (CM), systemic mastocytosis (SM) and mast cell sarcoma. Although CM is more common in children, it can also occur in adulthood, where it may remain confined to the skin or, more often, be associated with systemic disease. In SM, morphologically and immunophenotypically abnormal mast cells accumulate in the bone marrow and/or other extracutaneous organs [8,9]. Clinical manifestations include mediator-related symptoms such as flushing, anaphylaxis, gastrointestinal complaints, and musculoskeletal involvement. SM may also present or further progress to advanced variants with organ dysfunction.

In parallel, mast cell activation syndromes (MCASs) comprise a heterogeneous group defined by recurrent episodes of systemic symptoms associated with the release of mast cell-derived mediators. MCAS is broadly classified into monoclonal (clonal or primary, often linked to mastocytosis), secondary (non-neoplastic, usually IgE-mediated) and idiopathic variants (without an identifiable underlying disease or mast cell clonality) [10,11].

As the diagnostic testing for HαT became available very recently in most countries as well as in Greece, the aim of our study was to describe the first case series of confirmed HαT in patients with mastocytosis or MCAS and to highlight how recognition of this germline biomarker may inform personalized clinical assessment and management.

## 2. Materials and Methods

### 2.1. Study Design, Patient Population, and Data Collection

This single-center observational retrospective study was conducted on eight patients with genetically confirmed HαT in the context of coexisting mast cell disorders, under regular follow-up at the National Center of Expertise for Mastocytosis and Mast Cell Disorders, Allergy Unit, University General Hospital “Attikon” in Athens, Greece. Patient inclusion took place between January 2025 and May 2025. Both newly referred patients and individuals already under follow-up due to a coexisting mast cell disorder were eligible for inclusion. The relatively short inclusion period reflects the recent availability of *TPSAB1* genotyping for HαT in Greece. Among the participants, two had a concomitant diagnosis of indolent SM (ISM), one had CM and the remaining five fulfilled the diagnostic criteria for MCAS [12]. The diagnosis of MCAS was established according to published consensus criteria. These included: (i) recurrent, severe systemic symptoms consistent with mast cell mediator release involving at least two organ systems; (ii) objective evidence of mast cell activation, defined as a significant transient increase in serum tryptase levels (≥20% above baseline plus 2 ng/mL) or other mast cell–derived mediators, during a period of increased symptoms; and (iii) a documented clinical response to medications that counteract mast cell mediator effects and/or suppress mast cell activation [10,12].

Baseline data included demographic characteristics (age and sex), *TPSAB1* genotyping and detailed clinical history, encompassing clinical phenotype, age at symptom onset, diagnosis of an underlying mast cell disorder, prior therapeutic interventions, and comorbidities. Laboratory investigations at baseline included a comprehensive laboratory workup and measurement of BST levels. In our study, the Mastocytosis Activity Score (MAS), a validated patient-reported outcome instrument specifically developed to assess symptom activity in adult patients with CM and ISM, was used. The MAS is a linearly transformed scale ranging from 0 to 100, with higher values indicating greater mast cell–mediator-related symptom burden and disease activity. Based on validation data, MAS values may be categorized as follows: 0–16 corresponds to mild, 17–28 to moderate, and ≥29 to severe disease activity [13]. Although the MAS has not been validated in HαT, it was selected due to the substantial overlap in mediator-related symptoms between HαT and mast cell disorders and the absence of disease-specific validated patient-reported outcome measures for HαT. The MAS was therefore applied as an exploratory outcome measure to assess changes in symptom burden over time.

Patients were prospectively assessed at baseline (week 0) and at 8 weeks. Four patients were already receiving mediator-targeted therapy at the time of inclusion. In these cases, baseline assessment was defined following a 7-day washout period prior to study entry. The MAS was subsequently reassessed after therapeutic adjustment to evaluate changes in symptom burden over time. Assessments included: (a) MAS in order to quantify mast cell-related symptom burden across multiple organ systems, and (b) documentation of therapeutic interventions, including H1-/H2-antihistamines, leukotriene receptor antagonists, mast cell stabilizers, systemic corticosteroids, and anti-IgE therapy [14].

Treatment decisions were individualized based on symptom burden and clinical presentation. All patients received twice-daily H1- and H2-antihistamines, reflecting the commonly used intensified regimen for mediator-related symptoms in mast cell disorders. Montelukast was added in cases of incomplete control despite maximal antihistamine therapy. Omalizumab was considered in patients with recurrent anaphylaxis or severe hypersensitivity reactions refractory to standard therapy, and in selected cases to facilitate safe initiation of venom immunotherapy (VIT). Clinical response was evaluated both quantitatively, using the MAS, and qualitatively through documentation of symptom frequency, intensity, and anaphylaxis recurrence.

This study was conducted in accordance with the principles of the Declaration of Helsinki and complied with Good Clinical Practice (GCP) guidelines. All data were collected and processed exclusively for clinical purposes in compliance with applicable data protection regulations, and no personally identifiable information was accessible to individuals outside the clinical care team.

### 2.2. HαT Methodology

Genotyping of *TPSAB1* and *TPSB2* was performed with the method of digital PCR using the Absolute Q instrument (Thermo Fisher Scientific, Waltham, MA, USA). Prior to dPCR analysis the extracted genomic DNA was incubated with the restriction endonuclease BamHI (New England Biolabs, Hitchin, Hertfordshire, UK) to ensure that copies that are close to each other would not be partitioned in the same microchamber. For the dPCR reaction the Absolute Q Universal DNA MasterMix (Thermo Fisher Scientific, Waltham, MA, USA) was utilized, as well as primer/probe sets (Eurofins Genomics, Ebersberg, Germany) that capture the α and β isoforms from *TPSAB1* and *TPSB2* as described by Lyons et al. [4]. In order to accurately measure α-and β-tryptase copy numbers, the *AP3B1* gene was used as a reference with primers and probe sequences (Eurofins Genomics, Ebersberg, Germany) as previously described [15]. In order to ensure reproducibility, the extracted DNA was always measured using the Quawell 5000 Uv-vis spectrophotometer (Quawell Technology Inc., San Jose, CA, USA) prior to the assay to confirm adequate DNA purity and concentration, and each sample was run in triplicate using the same amount of DNA per reaction.

Regarding the QC criteria, the Absolute Q system (Thermo Fisher Scientific, Waltham, MA, USA) is calibrated to contain a QC channel where the passive reference dye (ROX™ dye; Thermo Fisher Scientific, Waltham, MA, USA) for each microchamber must be within a certain range defined by the manufacturer; otherwise it is automatically rejected and not included in the analysis by the software (QuantStudio Absolute Q Digital PCR Software, version 6.3; Life Technologies Corporation, Pleasanton, CA, USA). The microchambers in the MAP16 plates that are used for the reactions are 20,480 per array, and for each sample more than 20,000 must be filled to confidently declare that the vast majority of the sample was used for the dPCR reaction, which serves as another quality criterion.

For control DNA we used digested CEPH 1347-02 (Thermo Fisher Scientific, Waltham, MA, USA), which is a well-characterized diploid DNA commonly used as control in copy number variation studies to evaluate the assay’s performance, including reproducibility and precision in copy number determination. Thresholds were determined manually according to the aforementioned control, and adjusted per run after inspecting the 1D/2D scatter plots, so as to observe distinct clusters; those high and low in fluorescence, and assessed so as to not have intermediate “rain”.

### 2.3. Statistical Analysis

Data are presented as numbers and percentages for categorical parameters and as the mean ± standard deviation (SD) or median (range), as appropriate. Comparisons between repeated measurements within the same group (e.g., baseline vs. week 8) were conducted using the paired Student’s *t*-test, following confirmation of the normal distribution of the differences. A *p*-value < 0.05 was considered as statistically significant. All statistical analyses were performed using IBM SPSS-24 (Armonk, NY, USA). Graphs and visualizations were generated using GraphPad Prism version 10.4.2 (GraphPad Software, San Diego, CA, USA).

## 3. Results

### 3.1. Patient Characteristics

We identified eight patients with a confirmed diagnosis of HαT, established through *TPSAB1* genotyping [4]. Baseline patient characteristics are presented in Table 1. The mean age at the time of HαT diagnosis was 53.9 ± 12.0 years (range, 37–72).

The median BST level was 16.2 ng/mL (range, 14.3–51.2). Regarding *TPSAB1* genotypic distribution, the following proportions were observed: 62.5% 3α:2β and 37.5% 2α:3β. Among the study population, two patients had an established diagnosis of ISM, one of CM and five met the criteria for diagnosis of MCAS [16,17].

### 3.2. Mast Cell Disorders and Basal Serum Tryptase

**Figure 1:**
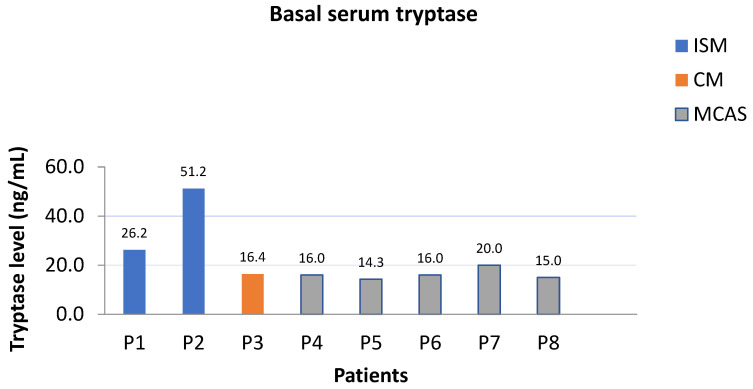
Basal serum tryptase levels by diagnostic subtype (ISM, CM, MCAS).

Among the eight patients referred to our center, six had a documented history of severe anaphylactic reactions, classified as grade III according to the Brown classification, while the remaining two were diagnosed with mastocytosis in the skin (MIS). In adults with skin lesions who did not undergo a complete staging with bone marrow analyses, the provisional diagnosis of MIS is appropriate. All patients exhibited elevated basal serum tryptase levels (>11.4 ng/mL) (Figure 1). Further diagnostic workup included molecular genetic testing for HαT through droplet digital PCR [4], as well as analysis for the *KIT* (receptor tyrosine kinase) D816V mutation, which was performed exclusively on peripheral blood samples [18].

Bone marrow biopsy was conducted in both patients with MIS and in one patient with peripheral blood detection of the *KIT* D816V mutation. As a result, two patients were diagnosed with SM, while one was confirmed to have CM [19,20]. Notably, the two SM cases fulfilled the diagnostic criteria for ISM, as neither B-findings (indicative of high disease burden) nor C-findings (organ damage attributable to mast cell infiltration) were identified [7].

In our cohort, SM diagnoses were observed in patients with higher BST levels (>20 ng/mL), whereas patients with BST levels ranging from 14 to 20 ng/mL demonstrated no evidence of systemic involvement. This finding is biologically plausible, as patients with concomitant SM and HαT harbor two independent factors associated with elevated BST levels, thereby increasing the likelihood of higher BST values.

### 3.3. Clinical Manifestations

Detailed individual clinical characteristics of patients with HαT are presented in Table 2. Regarding clinical manifestations, functional gastrointestinal complaints were the most frequently reported, including gastroesophageal reflux symptoms (7/8, 87.5%) and features consistent with irritable bowel syndrome (4/8, 50%). Cutaneous symptoms were present in 5/8 patients (62.5%), mainly flushing and pruritus, either occurring spontaneously or triggered by minor mechanical stimuli. Neuropsychiatric manifestations were likewise frequently observed, with sleep disruption reported in 5/8 (62.5%) and mood alterations in 4/8 (50%). Constitutional symptoms such as headache and arthralgia were each reported in 4/8 patients (50%). Finally, symptoms suggestive of autonomic dysfunction, including orthostatic hypotension and palpitations, were observed in 3/8 patients (37.5%) (Table 3).

Notably, anaphylactic reactions were reported in 6/8 patients (75%). Among these, 4/6 (66.7%) were attributed to drug exposure. Specifically, amoxicillin was implicated in 2/6 (33.3%) cases, omeprazole in 1/6 (16.7%), and parecoxib in 1/6 (16.7%). Both patients with amoxicillin-induced anaphylaxis and the patient who experienced anaphylaxis following parecoxib administration had a prior history of idiopathic anaphylaxis, while the patient who reacted to omeprazole had also experienced anaphylaxis after thiocolchicoside exposure. The remaining 2/6 anaphylactic reactions (33.3%) were triggered by Hymenoptera stings, with one patient experiencing recurrent episodes following honeybee stings and the other after repeated exposure to common wasp stings. All episodes were life-threatening and classified as grade III according to the Brown anaphylaxis severity classification [21]. Taken together, these findings highlight a high burden of multisystem mediator-related symptoms and a substantial prevalence of anaphylaxis among patients with HαT and coexisting mast cell disorders.

### 3.4. Mastocytosis Activity Score (MAS)

Although the MAS has not been evaluated in HαT [13], patients were asked to complete it at baseline and 8 weeks after treatment initiation, given the substantial overlap in clinical symptomatology between HαT and mastocytosis [1,4]. At baseline, the mean MAS was 27.25 ± 7.40 (range, 16–38), reflecting a high symptom burden among the evaluated patients. Following 8 weeks of treatment, the mean MAS decreased to 18.25 ± 6.48 (range, 12–27), indicating a substantial reduction in symptom severity across the study. In this exploratory analysis, the observed mean change in MAS was 9.00 points (95% CI: 6.17–11.83). These results are presented descriptively.

In addition, a categorical shift in MAS severity classification was observed. At baseline, 62.5% (5/8) of patients had moderate disease activity (MAS 17–28) and 37.5% (3/8) were classified as severe (MAS ≥ 29), while none were in the mild range. After 8 weeks of treatment, 62.5% (5/8) of patients had improved to the mild category and the remaining 37.5% (3/8) were classified as moderate, with no patients persisting in the severe category. Importantly, this improvement was not limited to patients with coexisting SM or CM (3/8), but was also observed in those diagnosed with MCAS (5/8), highlighting the consistent clinical benefit of mediator-targeted therapy across distinct mast cell-associated phenotypes within the HαT population.

All patients were initiated on high-dose antihistamine therapy targeting both H1- and H2-receptors, administered twice daily, as first-line symptomatic treatment. In patients with recurrent anaphylactic episodes, the inciting trigger was identified when possible and strict avoidance was advised. VIT was initiated in two patients with confirmed Hymenoptera venom allergy. Omalizumab was added in three patients with idiopathic anaphylaxis who continued to experience recurrent episodes despite maximal antihistamine therapy and montelukast, and in a fourth patient with honeybee venom allergy and no evidence of systemic mastocytosis, who developed breakthrough reactions during the initial phase of VIT. After an 8-week course of omalizumab pre-treatment, VIT was successfully reintroduced and was thereafter well tolerated [22]. Following omalizumab initiation, all four patients exhibited a marked reduction in anaphylactic events, with overall improvement in their symptom burden. Consistent with this response, cutaneous and constitutional symptoms improved in all affected patients, while gastrointestinal manifestations showed a favorable response in several cases (6/8). In contrast, neuropsychiatric and autonomic symptoms remained largely refractory. Overall, treatment initiation was associated with a consistent reduction in mediator-related symptom burden, as reflected by both quantitative improvement in MAS and categorical shifts toward milder disease activity.

## 4. Discussion

The severity and heterogeneity of clinical manifestations in HαT vary considerably among affected individuals. Moreover, the clinical phenotype often overlaps with other disorders, posing significant challenges to accurate diagnosis [4,23]. In our study we report the clinical findings and the genetic profile of the first eight Greek patients with HαT and concomitant mast cell disorders.

Our findings are consistent with previous cohorts reporting gastrointestinal, cutaneous, neuropsychiatric, and autonomic manifestations as common features of symptomatic HαT [24]. Moreover, prior studies, including that of Greiner et al., have reported greater mediator-related symptom burden in patients with mastocytosis and HαT compared with those without HαT [25]. In our cohort, similar symptom patterns were observed across patients with different mast cell disorders, including MCAS.

Beyond chronic mediator-related manifestations, anaphylaxis emerged as a major clinical concern in our study. The majority of reactions were drug-induced, while Hymenoptera venom also accounted for life-threatening episodes. This distribution aligns with recent data by von Bubnoff et al., who reported drugs as the predominant elicitors of anaphylaxis in symptomatic HαT patients, with Hymenoptera stings remaining a recognized cause of severe reactions [24]. Importantly, Lyons et al. demonstrated that HαT constitutes a heritable genetic risk factor for severe anaphylaxis, both in the general population and in patients with systemic mastocytosis [6]. Our data extend this concept by demonstrating an association between HαT and a higher frequency of anaphylactic reactions in patients with MCAS, underscoring that its clinical impact is not confined to mastocytosis. Taken together, current evidence indicates that HαT substantially enhances susceptibility to anaphylaxis across diverse triggers, highlighting the need for heightened vigilance in clinical practice.

Considering the variable expressivity of HαT, therapeutic considerations should extend beyond serum tryptase levels and be individualized. In the Brigham and Women’s Hospital experience of 101 HαT patients, most individuals were managed with H1-and H2-antihistamines, which provided only partial relief of mediator-related symptoms [26]. Likewise, in our series, cutaneous and constitutional symptoms improved substantially with high-dose antihistamines, while neuropsychiatric and autonomic features were largely refractory. With respect to omalizumab, Giannetti et al. reported clinical improvement in approximately 80% of patients treated for recurrent anaphylaxis [26]. Consistently, in our study omalizumab was introduced in three patients with idiopathic anaphylaxis, and in one patient with honeybee venom allergy, where pre-treatment with omalizumab enabled safe re-initiation of VIT after recurrent breakthrough reactions, suggesting a potential adjunctive role in selected high-risk cases. This therapeutic choice was guided by the anti-IgE mechanism of omalizumab, which leads to downregulation of FcεRI expression on mast cells and basophils and has been associated with clinical benefit in mast cell–mediator–driven symptoms and prevention of anaphylaxis. Whereas previous reports have described breakthrough reactions during VIT mainly in patients with systemic mastocytosis [27], our observation in a patient with MCAS and HαT suggests that this risk may extend beyond mastocytosis.

From a personalized medicine perspective, HαT represents a germline biomarker that may contribute to a more nuanced understanding of clinical heterogeneity among patients with mast cell disorders. Notably, all patients in the present cohort carried a *TPSAB1* gene duplication, resulting in limited genotypic heterogeneity and consequently limiting the ability to explore meaningful genotype–phenotype associations. *TPSAB1* copy number variation was observed in a subgroup of patients presenting with frequent anaphylactic episodes, prominent gastrointestinal and neuropsychiatric manifestations, and a high mediator-related symptom burden. Our findings show that treatment response—particularly improvement in MAS and benefit from omalizumab—varied across clinical phenotypes, supporting an individualized therapeutic approach based on symptom clusters rather than disease category alone. Incorporating HαT genotyping into diagnostic pathways therefore allows earlier identification of high-risk individuals, facilitates tailored prevention strategies (including venom immunotherapy pre-treatment), and refines therapeutic decision-making within the broader framework of precision medicine. Importantly, these observations highlight that recognition of HαT may assist clinicians in interpreting disproportionate clinical severity and heterogeneity among patients with otherwise similar mast cell-related diagnoses, thereby supporting patient-specific management decisions in routine clinical practice.

Emerging clinical evidence suggests a potentially increased burden of autoimmune comorbidities among individuals with HαT. In our cohort, two (25%) patients had comorbid psoriasis, suggesting a markedly increased prevalence compared to general population estimates, which range between 2% and 3% [28]. Notably, one of these patients was also diagnosed with ulcerative colitis, potentially reflecting overlapping immune dysregulation. In addition, one patient presented with alopecia areata. These findings underscore the need for further investigation into autoimmune associations in the context of HαT [29,30].

Key limitations of our study include the small sample size, its single-center design, the absence of a control group, and potential selection bias due to recruitment at a center of expertise within a tertiary care hospital. Furthermore, the use of MAS to assess symptom burden in this cohort represents an additional limitation, as this instrument has not been validated for patients with HαT. Currently, no disease-specific or validated symptom assessment instrument exists for HαT, which hinders standardized evaluation and cross-study comparisons of clinical burden. In addition, the presence of comorbid mast cell disorders among the study population—including SM (2/8), CM (1/8) and MCAS (5/8)—may have contributed to the overall symptom burden. Importantly, these findings reflect early, short-term observations over an 8-week follow-up period following therapeutic adjustments within the study framework. Given the absence of a control group, the heterogeneity of concomitant interventions, and the short duration of follow-up, the observed improvement in symptom burden cannot be attributed to any individual therapeutic component, nor can causality be inferred. Future multicenter studies with larger patient cohorts and longer follow-up are warranted to further delineate the clinical spectrum of HαT and to enable the development and validation of disease-specific outcome measures.

Despite the limitations, this study contributes valuable real-world evidence on the clinical phenotype of HαT, a condition that remains underrepresented in the current literature. To our knowledge, this is the first clinical characterization of patients with genetically confirmed HαT in a Greek cohort. All patients were evaluated at a nationally designated center of expertise for mast cell disorders, ensuring diagnostic precision and consistency in clinical assessment and management. Furthermore, the detailed documentation of multisystem symptoms and comorbidities, based on structured clinical assessments, allows for meaningful comparison with previously published international cohorts. Finally, the early clinical improvement following initiation of mediator-targeted therapy further supports the potential role of symptomatic treatment in alleviating the multisystem burden of HαT. This contributes to the growing body of literature on HαT and supports the recognition of its diverse and overlapping clinical manifestations.

## 5. Conclusions

The discovery of HαT has provided critical insights into the genetic regulation of tryptase, elucidating key mechanisms underlying its expression and secretion in both physiological and pathological contexts. This has contributed substantially to our broader understanding of mast cell biology and its relevance to a spectrum of clinical conditions. *TPSAB1* testing should be regarded as a clinically relevant genetic marker that has expanded our understanding of interindividual variability in mast cell disorders. Beyond its diagnostic utility, *TPSAB1* genotyping may provide complementary genetic information that aids in the clinical interpretation of mediator-related symptoms, including anaphylactic reactions. Integrating HαT evaluation into clinical pathways may therefore facilitate a more individualized clinical approach.

Despite these advances, the potential role of HαT in the pathophysiology of mast cell activation and its contribution to the etiology of mast cell disorders remain incompletely understood. Accordingly, further mechanistic and clinical investigations are essential to elucidate the extent to which HαT may act as a driver or modifier of mast cell-mediated diseases.

## Figures and Tables

**Table 1:** Summary of Clinical and Genetic Characteristics of Patients. ISM: Indolent Systemic Mastocytosis, CM: Cutaneous Mastocytosis, MCAS: Mast Cell Activation Syndrome.

Patient Characteristics	Hereditary Alpha Tryptasemia (*n* = 8)
Age, mean ± SD (range)	53.9 ± 12.0 years (37–72)
Sex, male; N (%)	5 (62.5%)
Basal serum tryptase, median (range)	16.2 ng/mL (14.3–51.2)
*TPSAB1* genotype	
3α:2β	5 (62.5%)
2α:3β	3 (37.5%)
Diagnosis	
ISM	2 (25%)
CM	1 (12.5%)
MCAS	5 (62.5%)

**Table 2:** Individual Clinical Characteristics of Patients with HαT.

Patient	Age (Years)	Sex	Diagnosis	BST (ng/mL)	*TPSAB1* Genotype	Key Comorbidities	Clinical Manifestations	Anaphylaxis History	Treatment
P1	49	Male	ISM	26.2	3α:2β	None	GERD symptoms, Arthralgia, Mood alteration	Common Wasp	H1-/H2-antihistamines, VIT
P2	57	Female	MCAS	15.0	2α:3β	Psoriasis, Ulcerative Colitis	Arthralgia, Headache, Flushing/pruritus, IBS, Sleep disruption, Autonomic dysfunction	Parecoxib, Idiopathic	H1-/H2-antihistamines, Montelukast, Omalizumab
P3	72	Male	CM	16.4	3α:2β	None	GERD symptoms, Headache, Flushing/pruritus, Sleep disruption, Autonomic dysfunction	None	H1-/H2-antihistamines
P4	52	Female	MCAS	16.0	3α:2β	Alopecia Areata	GERD symptoms, Flushing/pruritus, IBS, Sleep disruption, Mood alteration	Amoxicillin, Idiopathic	H1-/H2-antihistamines, Montelukast, Omalizumab
P5	39	Male	MCAS	14.3	2α:3β	None	GERD symptoms, Headache, IBS, Sleep disruption, Autonomic dysfunction	Honeybee	H1-/H2-antihistamines, Omalizumab, VIT
P6	67	Female	MCAS	16.0	3α:2β	None	GERD symptoms, Headache, Flushing/pruritus, IBS, Sleep disruption, Mood alteration	Amoxicillin, Idiopathic	H1-/H2-antihistamines, Montelukast, Omalizumab
P7	54	Male	MCAS	20.0	3α:2β	Psoriasis	GERD symptoms, Arthralgia, Mood alteration	Omeprazole, thiocolchicoside	H1-/H2-antihistamines
P8	37	Male	ISM	51.2	2α:3β	None	GERD symptoms, Arthralgia Flushing/pruritus	None	H1-/H2-antihistamines

BST: Basal Serum Tryptase, ISM: Indolent Systemic Mastocytosis, CM: Cutaneous Mastocytosis, MCAS: Mast Cell Activation Syndrome, GERD: Gastroesophageal Reflux Disease, IBS: Irritable Bowel Syndrome.

**Table 3:** Clinical Features of Patients in the National Center of Expertise for Mastocytosis and Mast Cell Disorders (*n* = 8).

Manifestation	Reported Frequency (%)
Gastrointestinal symptoms	
Chronic gastroesophageal reflux symptoms	87.5%
Irritable bowel syndrome (Rome III)	50%
Cutaneous symptoms	
Flushing/Pruritus	62.5%
Neuropsychiatric symptoms	
Sleep disruption	62.5%
Mood alterations	50%
Systemic hypersensitivity reactions	
Drug-induced	50%
Venom hypersensitivity reaction	25%
Constitutional symptoms	
Headache/Body pain	50%
Arthralgia	50%
Autonomic dysfunction	
Orthostatic hypotension/Palpitations	37.5%

## Data Availability

The data supporting the findings of this study are derived from patients followed at the Center of Expertise of the European Competence Network on Mastocytosis based at the Allergy Unit, University General Hospital “Attikon”, Greece. Due to patient privacy and confidentiality regulations, the raw data are not publicly available. However, anonymized data may be provided by the corresponding author upon reasonable request.

## Abbreviations

The following abbreviations are used in this manuscript:

BST	basal serum tryptase
CM	cutaneous mastocytosis
HαT	hereditary alpha-tryptasemia
IA	idiopathic anaphylaxis
ISM	Indolent Systemic Mastocytosis
MAS	Mastocytosis Activity Score
MCAS	mast cell activation syndrome
MIS	mastocytosis in the skin
SM	systemic mastocytosis
VIT	venom immunotherapy
